# Interacting Epidemics and Coinfection on Contact Networks

**DOI:** 10.1371/journal.pone.0071321

**Published:** 2013-08-08

**Authors:** M. E. J. Newman, Carrie R. Ferrario

**Affiliations:** 1 Center for the Study of Complex Systems and Department of Physics, University of Michigan, Ann Arbor, Michigan, United States of America; 2 Department of Pharmacology, University of Michigan, Ann Arbor, Michigan, United States of America; Umeå University, Sweden

## Abstract

The spread of certain diseases can be promoted, in some cases substantially, by prior infection with another disease. One example is that of HIV, whose immunosuppressant effects significantly increase the chances of infection with other pathogens. Such coinfection processes, when combined with nontrivial structure in the contact networks over which diseases spread, can lead to complex patterns of epidemiological behavior. Here we consider a mathematical model of two diseases spreading through a single population, where infection with one disease is dependent on prior infection with the other. We solve exactly for the sizes of the outbreaks of both diseases in the limit of large population size, along with the complete phase diagram of the system. Among other things, we use our model to demonstrate how diseases can be controlled not only by reducing the rate of their spread, but also by reducing the spread of other infections upon which they depend.

## Introduction

Two diseases circulating in the same population of hosts can interact in various ways. One disease can, for instance, impart cross-immunity to the other, meaning that an individual infected with the first disease becomes partially or fully immune to infection with the second [1], [2]. A contrasting case occurs when infection with one disease *increases* the chance of infection with a second. A well-documented example is HIV, which, because of its immunosuppressant effects, increases the chances of infection with a wide variety of additional pathogens. Other examples include syphilis and HSV–2, the presence of either of which can substantially increase the chances of contracting, for example, HIV [3]–[6]. In a non-disease context, similar phenomena also arise in the epidemic-like spread of fashions, fads, or ideas through a population. There are, for instance, many examples of products whose adoption or purchase depends on the consumer already having adopted or purchased another product. The purchase of software or apps for computers or phones, for instance, requires that the purchaser already own a suitable computer or phone. In cases where adoption of products spreads virally, by person-to-person recommendation, a “coinfection” model of adoption may then be appropriate.

In this paper we study mathematically the behavior of infections that promote or are promoted by other infections in this way. We consider a model coinfection system with two diseases, both displaying susceptible–infective–recovered (SIR) dynamics [7], [8], in which any individual may contract the first disease if exposed to it, but the second disease can be contracted only by an individual previously infected with the first. This is a simplification of the more general situation in which absence of the first disease decreases the chance of infection with the second but does not eliminate it altogether. It is, however, a useful simplification, retaining many qualitative features of the more general case, while also allowing us to solve for properties of the model exactly. Following previous work on competing pathogens [2], we assume the spread of our two diseases to be well temporally separated, the first disease passing completely through the population before the second one strikes, although arguments of [9] suggest that this assumption could be relaxed without significantly altering the results.

The choice of SIR dynamics for our model appears at first to be less appropriate for a disease like HIV, from which sufferers do not normally recover. However, HIV is mainly infective during its primary stage–the first few weeks of infection–after which it enters an asymptomatic stage where probability of transmission is much lower [10], [11]. The “recovered” state of our model can mimic this behavior, at least for some populations with HIV.

Following the description outlined above one can easily write down a fully-mixed compartmental model of our interacting diseases in the style of traditional mathematical epidemiology, but the results are essentially trivial. The first disease spreads through the population according to ordinary SIR dynamics, then the second spreads in the subset of individuals infected by the first, but otherwise again following ordinary SIR dynamics. No qualitatively new behaviors emerge.

Real diseases, however, are not fully mixed. Rather, they spread over a network of physical contacts between individuals, whose structure is known to have a substantial impact on patterns of infection [12]–[14]. As we will demonstrate, the spread of our two interacting diseases shows a number of interesting behaviors once the presence of such an underlying contact network is taken into account.

## The model

We study a network-based model of interacting pathogens spreading through a single population, which we solve exactly using the cavity method of statistical physics. From our solution we are able to calculate the expected number of individuals infected with each of the two diseases as a function of disease parameters, as well as the epidemic thresholds and complete phase diagram of the system.

Our model consists of a network of 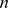 nodes, representing the individuals in the modeled population, connected in pairs by edges representing their contacts. The spread of the first disease through the network is represented by an SIR process in which all individuals start in the susceptible (S) state except for a single, randomly chosen individual who is in the infective (I) state–the initial carrier of the first disease. Infectives recover after a certain time 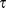, which we take to be constant, but while infective they have a fixed probability 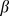 per unit time of passing the disease to their susceptible neighbors. The probability of the disease being transmitted in a short interval of time 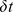 is thus 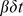 and the probability of it not being transmitted is 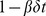. Thus the probability of not being transmitted during the entire time interval 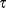 is
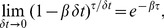
(1)and the total probability of being transmitted, the so-called infectivity or transmissibility 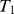 for the first disease, is



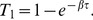
(2)


We will consider this quantity to be an input parameter to our theory.

Once the first disease has passed through the population, leaving every member of the population in either the susceptible or the recovered state with no infectives remaining, then the second disease starts to spread, but with the important caveat that it can spread only among those who have previously contracted, and then recovered from, the first disease, a state that we will denote 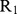. The second disease spreads among these individuals again according to an SIR process, and we will explicitly allow for the possibility that the second disease has a different transmissibility 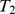 from the first. Note however that the second disease is still transmitted over the same contact network as the first, which can lead to nontrivial correlations between the probabilities of infection with the two diseases. Because the network is assumed the same for both diseases our model is primarily applicable to pairs of diseases having the same mode of transmission–two airborne diseases, for example, or two sexually transmitted diseases.

When the second disease has passed entirely through the system, every member of the population is left in one of three states: susceptible (S), meaning they have never contracted either disease; infected by and recovered from the first disease, but uninfected by the second (denoted 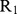); or infected by and recovered from both diseases (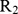). Note that there are no individuals who contract the second disease but not the first, since the first is a necessary condition for infection with the second. The number of individuals in the 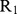 and 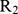 states tell us the total number who contracted each of the two diseases, and hence the size of the two outbreaks. As we will see, there is a nontrivial phase diagram describing the variation of these numbers with the transmissibilities 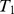 and 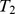 of the diseases.

To fully define our model we need also to specify the structure of the network of contacts over which the diseases spread. Many choices are possible, including model networks or networks based on empirical data for real contacts. In this paper, we employ one of the most widely used model networks as the substrate for our calculations, the so-called configuration model [15], [16]. The configuration model is a random graph model in which the degrees of nodes–the number of connections they have to other nodes–are free parameters that may be chosen from any distribution. Numerous studies in recent years have shown the degrees of nodes to have a large impact on the structure and behavior of networked systems [17]–[19], so a model that does not allow for varying degrees would be missing one of the most important of network properties. In respects other than this, however, the configuration model assumes random connections between nodes, which, it turns out, makes the network simple enough that we can solve exactly for the behavior of our two-disease system upon it.

The configuration model is completely specified by giving the number 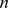 of nodes in the model network, which we will assume to be large, and the probability distribution of the degrees. The latter is parametrized by the fraction 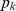 of nodes that have degree 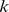, for 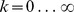. For instance, one might specify the degrees to have a Poisson distribution:
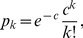
(3)where 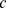 is the average degree in the network as a whole.

An alternative way of thinking about 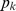 is as the probability that a randomly chosen node has degree 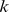. In our calculations we will also need to consider randomly chosen edges and ask what the probability is that the node at one end of such an edge has degree 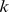. It is clear that this probability cannot in general be equal to 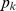. For instance, there is no way to follow an edge and reach a node of degree zero, even if degree-zero nodes exist in the network. So nodes at the end of an edge must have some other distribution of degrees. In fact, the relevant quantity for the purposes of this paper will be not the degree of the node at the end of an edge, but the degree minus one, which is the number of edges attached to the node other than the edge we followed to reach it. This number, often called the excess degree, has distribution
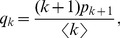
(4)where 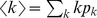 is the average degree in the network [18]. The quantity 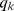 is called the excess degree distribution and both 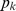 and 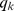 will play important roles in the developments here.

Because they will be useful later, we also define probability generating functions for the two distributions:
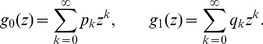
(5)


In what follows, we will assume we know the degree distribution  of our network, and hence that we know also the excess degree distribution, from Eq. (4), and the two generating functions, Eq. (5).

### Solution for the Number of Individuals Infected

We can solve exactly for the expected number of individuals infected by our two diseases on configuration model networks with arbitrary degree distributions. The calculation for the first disease is the simpler of the two, so we start there. This part of the solution follows closely the outline of our previous presentations in [13], [20].

**Figure 1:**
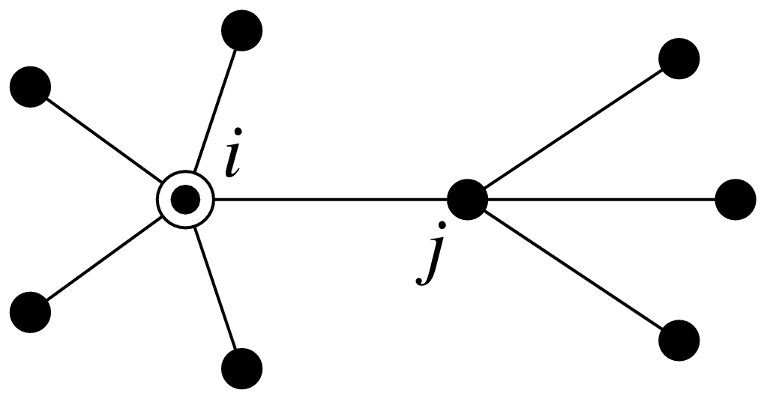
Probability of infection of a node with either one or both of the two diseases. We calculate the probability of infection of node 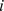 (circled) by first calculating the probability that a neighbor 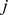 is infected. We must account separately for cases in which 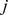 caught the first disease from 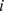 itself or from another of its neighbors, since these two cases have different implications for the spread of the second disease.

Consider Fig. 1, which depicts the neighborhood of a typical node 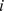 somewhere in the network, and let us calculate the average probability 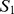 that such a node will ever be infected by disease 1. To do this we first consider the probability that a neighbor of 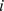, call it node 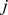, will be infected by disease 1 if 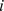 is removed from the network. Let us denote this latter probability by 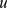.

The removal of node 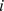 is a crucial element of our calculation. Some neighbors of 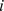 may be infected by 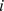 itself, but such a neighbor cannot then infect 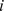 back, since 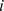 by definition already has the disease. Thus in calculating the probability of 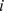
′s infection we need to discount such processes and count only neighbors of 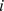 who were “externally infected,” meaning they were infected by one of their neighbors other than 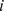. A simple way to achieve this is to remove 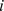 entirely from the network.

Once 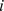 is removed from the network, the infection states of 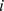
′s neighbors become statistically independent–the infection of one makes the infection of others no more or less likely. This is a particular property of configuration model networks in the limit of large network size. Such networks contain closed loops of edges that could in principle induce correlations between the states of nodes, but in the limit of large size the length of these loops diverges and the correlations vanish. The statistical independence between the neighbors of 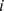 is the crucial property that makes exact calculations possible for our model.

If we know the value of 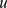, the probability of external infection of a neighboring node of 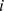, then the value of 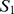, the average probability of infection of 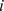 itself, is readily calculated as follows. A neighbor 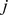 of node 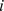 is infected with probability 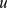 and transmits that infection to 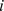 with probability equal to the transmissibility 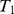, for an overall probability of infection 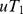. Then the probability of 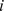 not being infected by 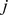 is 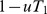 and the probability of 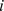 not being infected by any of its neighbors is 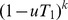 if it has exactly 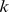 neighbors–the statistical independence of the neighbor states means that the probability for all 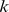 neighbors is just the probability for a single one to the 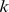
th power. Now averaging this quantity over the degree distribution 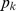, we find the mean probability that 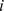 is not infected to be
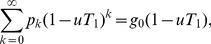
(6)where 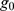 is the generating function for the degree distribution defined in Eq. (5). Then the probability that 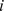 is infected is



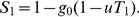
(7)It remains for us to find the value of 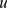, which we can do by an analogous calculation. The probability that neighboring node 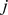 is not (externally) infected takes the form 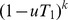, just as for node 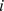, except that 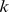 now represents the number of external neighbors of 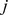, neighbors other than 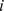. This is the number we previously called the excess degree of 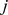, and it is distributed according to the excess degree distribution of Eq. (4). Averaging over this distribution, the mean probability that 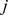 (or any neighbor node) is not infected is given by
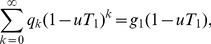
(8)where 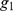 is the generating function for the excess degree distribution. Then the probability that 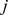 *is* externally infected is



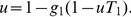
(9)Between them, Eqs. (7) and (9) allow us to solve for the average probability of infection of a node by disease 1: we first solve the self-consistent condition (9) for the value of 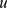, then we substitute the result into (7) to get the value of 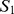. Note, moreover, that if 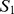 is the probability of infection, then 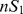 is the expected number of individuals infected with disease 1, so this calculation also gives us the expected size of the outbreak of disease 1.

This calculation is an example of a cavity method, a technique commonly used in statistical physics for the solution of network and lattice problems. The word “cavity” refers to the node 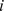 which is removed, leaving a hole or cavity in the network. The calculation above is a particularly simple example of the cavity method. The calculation of the spread of the second disease, however, which also makes use of the cavity method, is less simple.

Consider then the equivalent calculation for the second disease, in which we calculate the average probability that a node is infected with the second disease, the first disease having already spread through the system. An important point to recognize is that the subset of nodes through which the second disease spreads, which is the subset that was previously infected with the first disease, does not itself form a configuration model network. This is made clear for instance by the fact that the subset in question is connected–it forms a single network component–which is not true in general of configuration model networks [16]. As a result, our cavity method calculation is not the same for disease 2 as it was for disease 1, being rather more delicate. In particular, as we will see, the cavity node 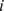 must now be removed for some parts of the calculation but not for others. We will break the calculation down into a number of steps.

First, when disease 1 spreads, node 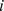 either gets infected (with probability 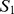 given by Eq. (7) above) or it does not (with probability 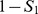). If it is not infected then it cannot later be infected with disease 2, and hence our calculation is finished–we need go no further. In all subsequent steps, therefore, we will assume that 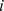 has been infected with (and has then recovered from) disease 1, a state that we previously denoted 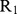.

Suppose that node 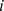 has degree 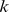. Let us ask what the value is of the probability 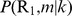 that it was infected with disease 1 and also has exactly 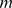 neighbors who were externally infected with disease 1, meaning that they were infected by any of *their* neighbors other than 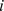
–see Fig. 1 again.

We note that 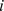 must have contracted disease 1 from one of its externally infected neighbors and the probability of this happening is
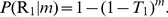
(10)


Also, since the probability of a neighbor’s external infection with disease 1 is 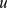 by definition, the probability of having 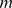 externally infected neighbors is
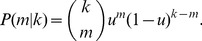
(11)


Combining these expressions, we have
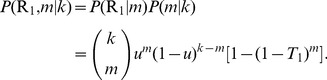
(12)


The number 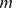, however, does not reflect the total number of 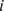
′s neighbors who have had disease 1 because, in addition to those infected externally as above, some number 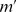 of the 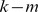 remaining nodes may also have been infected directly by 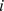 itself. (This is the part of the calculation in which 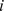 must not be considered removed from the network.) Given that 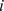 has had disease 1, the probability of such a direct infection for a neighbor of 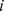 is just 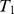 and hence
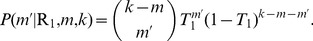
(13)


Combining Eqs. (12) and (13) we have
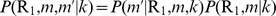


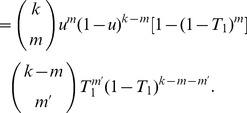
(14)


And, multiplying by the probability 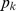 of having degree 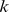, summing over 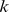, then dividing by the prior probability 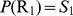 of contracting disease 1, we get
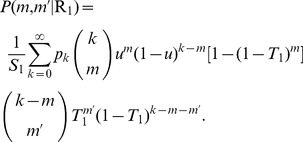
(15)


This quantity represents the probability that a node 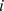 that has had disease 1 has 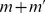 neighbors who have also had disease 1, of whom 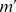 were infected by 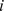 itself and the remaining 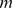 contracted their infections from other sources.

We can usefully encapsulate this rather complicated expression in a double generating function 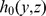 for the number of infected neighbors of 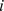 thus:
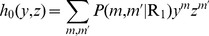


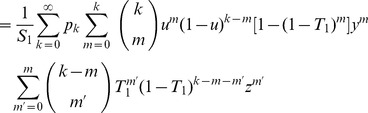


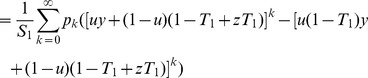


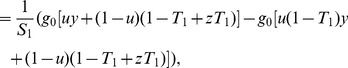
(16)where 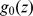 is the generating function for the degree distribution defined in Eq. (5). (As a check on this formula, we note that if we set 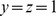 we should get 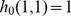. We leave it as an exercise for the particularly avid reader to demonstrate that this is indeed true.)

Given these results, the probability 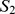 that node 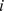 is infected with disease 2 given that it was previously infected with disease 1, is calculated as follows. Let 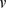 be the probability that a neighbor of node 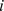 is externally infected with disease 2 (i.e., not via node 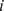) given that it has already been externally infected with disease 1. Then the probability that 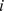 is infected with disease 2 by a neighbor that externally contracted disease 1 is 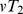 and if there are 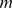 such neighbors in total then the probability of 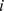 not contracting disease 2 from any of them is 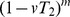.

Conversely, let 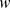 be the probability that a neighbor of 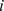 is externally infected with disease 2 given that it was *internally* infected with disease 1, meaning it was infected directly by node 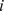. (As we will see in a moment, the probabilities 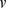 and 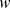 are not the same, so we must treat them separately.) Then the probability that 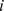 fails to contract disease 2 from any of the 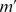 such nodes is 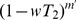.

Combining these results, the probability that 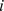 does not contract disease 2 at all is 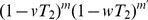 and the probability 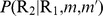 that it does is one minus this quantity. Averaging over 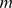 and 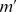, we find that
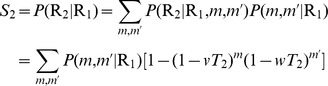


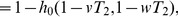
(17)where we have made use of the generating function 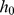 defined in Eq. (16).

This expression is the equivalent of Eq. (7) for the probability of infection with disease 2. It gives us the mean probability that an individual is infected with disease 2 given that it was previously infected with disease 1. Alternatively, 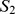 is the fraction of those individuals infected with disease 1 that also contract disease 2, 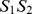 is the fraction of individuals in the entire network that contract disease 2, and 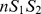 is the expected number of individuals with disease 2.

We have yet to calculate the values of the quantities 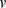 and 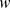, but these calculations are now quite straightforward. The calculation of 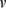 is the exact analog of the calculation we have already performed for 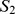. We calculate the probability that a neighbor of 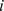 itself has 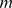 (or 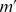) neighbors externally (internally) infected with disease 1, which is given by Eq. (15) but with 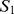 replaced with 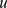 and 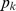 replaced with 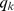. The generating function for this distribution is then the natural generalization of Eq. (16):
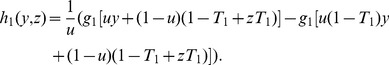
(18)


Then 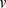 is the solution to the self-consistent condition
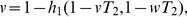
(19)which is analogous to Eq. (17).

The calculation of 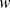 is a little trickier. Recall that 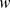 is the probability that 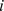
′s neighbor 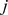 is externally infected with disease 2 given that it was *internally* infected with disease 1 (i.e., via node 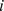). If 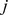 has exactly 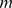 neighbors (other than 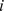) that were externally infected with disease 1, then the probability that all of them failed to infect 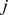 is 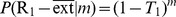, where the notation “
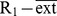
” denotes that 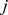 was not externally infected. If 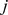 has excess degree 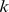 then 
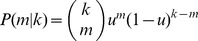
, and
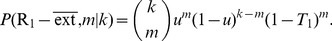
(20)


The number 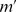 of neighbors of 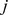 infected with disease 1 by 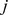 itself is distributed according to
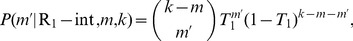
(21)where “
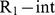
” denotes that 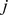 was internally infected. Noting that 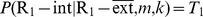 since 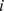 has presumptively had disease 1 and has probability 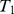 of having transmitted it to 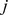 regardless of the values of 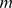 and 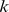, we have



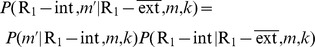


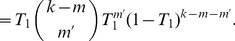
(22)


Multiplying Eqs. (20) and (22) and noting that 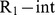 always implies 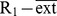, we get an expression for 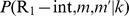. Then we multiply by 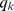 and sum over 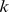 to get 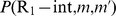, and divide by the prior probability 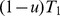 of being internally infected with disease 1 to get
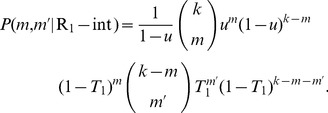
(23)


The generating function for this probability distribution is
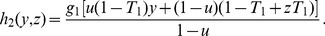
(24)


Finally, 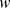 itself is given by the equivalent of Eq. (19):
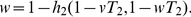
(25)


Our complete prescription for calculating the number of nodes infected with both diseases is now as follows. (1) We solve Eqs. (7) and (9) for 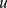 and 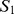; (2) we use the value of 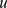 to solve Eqs. (19) and (25) for 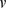 and 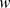, given the definitions of 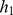 and 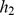 in Eqs. (18) and (24); (3) we substitute the resulting values into Eq. (17) to find 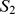.

As an added bonus, the quantities 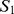 and 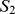 also tell us the probabilities of epidemic outbreaks of each of our two diseases. As discussed in Ref. [13], not all outbreaks of a disease reach a large fraction of the population. The infection process is stochastic and sometimes, by luck, a disease starting with a single initial carrier will not get passed to anyone else, or will get passed to only a few and then fizzle out. Other times it will take off and become an epidemic, and the probability of it doing this is exactly equal to the fraction of the network ultimately infected with the disease. Thus the probability of an epidemic outbreak of disease 1 is simply 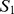, and the probability of an epidemic outbreak of disease 2 is 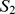 given that an outbreak of disease 1 already happened, or 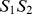 overall.

### Epidemic Thresholds

It is possible for either 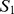 or 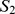 to be exactly zero, in which case there will under no circumstances be an epidemic of the corresponding disease. In general there will be threshold values of the transmission probabilities 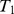 and 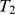 below which no epidemics occur and we can calculate the position of these epidemic thresholds from the equations given in the previous section.

First consider disease 1, which is the simpler of the two. The size of the outbreak of disease 1 falls to zero when 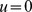, since this is the point at which the probability of a node catching the disease from its network neighbors vanishes. (We can confirm this directly by setting 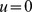 in Eq. (7), which gives 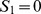 since 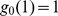.) The value of 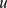 is given by Eq. (9). When we approach the epidemic transition from above, 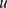 becomes small and we can expand the equation in powers of this small parameter as
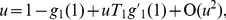
(26)where the prime denotes differentiation. But 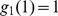 and the higher-order terms can be dropped in the limit as 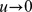, and hence we find the value of 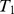 in this limit, which is by definition the epidemic threshold value 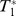, to be



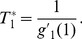
(27)This is a well known result which appears elsewhere in the literature [13].

For the second disease there are two ways in which the disease can fail to create an epidemic. The first is that disease 1 fails to create an epidemic, in which case disease 2 must also fail, since it depends on disease 1 for its propagation. The second is that disease 1 creates an epidemic, but the transmissibility of disease 2 is not high enough to create a second epidemic among the subset of the population infected with disease 1. Assuming we are in this second regime, the size of the second epidemic goes to zero when 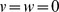 where 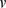 and 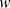 are the simultaneous solutions of Eqs. (19) and (25). Applying the same method as for disease 1, we consider a point slightly above the epidemic threshold, where 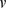 and 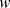 are small, and we expand in both to get
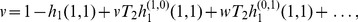
(28)

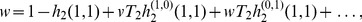
(29)where the superscript 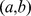 denotes differentiation of the generating functions with respect to their first and second arguments 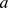 and 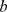 times respectively. Observing that 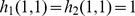 and neglecting the higher terms in the limit as we go to the epidemic transition, we have in matrix notation
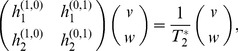
(30)where the derivatives are evaluated at the point 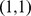. In other words 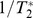 is an eigenvalue of the 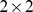 matrix on the left-hand side.

The eigenvalues of a general 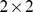 matrix are equal to 
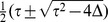
, where 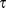 and 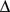 are the trace and determinant of the matrix. Making use of the definitions of 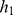 and 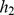 in Eqs. (18) and (24), we find the four derivatives appearing in our matrix to be
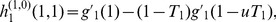
(31)

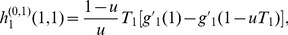
(32)

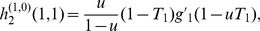
(33)

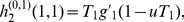
(34)which means



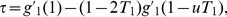
(35)

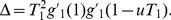
(36)


It remains only to determine which of the two eigenvalues gives the correct result for 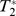. This can be done by setting 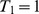, which gives 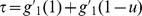 and 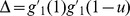, and hence the two eigenvalues are 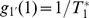 and 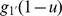. Logic dictates that the first eigenvalue must be the correct choice: when 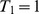 the second disease is spreading on the entire network and hence its epidemic threshold must fall at 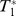. Thus, we find that
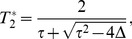
(37)where 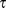 and 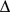 are given by Eqs. (35) and (36).

Notice that if we take the limit 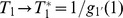 from above, which implies that 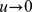, then we have 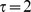 and 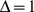 and hence 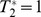. That is, when we are precisely at the epidemic threshold for the first disease, the threshold for the second disease is 1. We expect 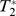 to be a monotone decreasing (or at least non-increasing) function of increasing 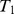 and when 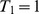 we have 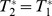 as shown above. So we expect 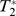 to be monotone decreasing in 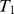 and 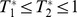 at all times.

Thus the epidemic threshold for disease 2 is never lower than the epidemic threshold for disease 1. The intuitive explanation of this result is that the constraint on disease 2, that it spread solely among individuals already infected with disease 1, only ever reduces the set of nodes it can spread on and hence makes it harder, never easier, for the disease to spread.

### Examples

As a concrete example of the results of the previous sections, consider interacting diseases spreading on a network with a Poisson degree distribution with mean degree 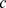, as in Eq. (3). This distribution presents a particularly simple case, because the excess degree distribution is equal to the ordinary degree distribution 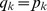 and their two generating functions are equal
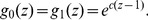
(38)


Thus 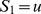 and 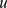 is a solution of
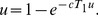
(39)


Similarly 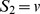 and 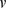 and 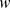 are solutions of Eqs. (19) and (25), though neither of the latter equations is very simple.

**Figure 2:**
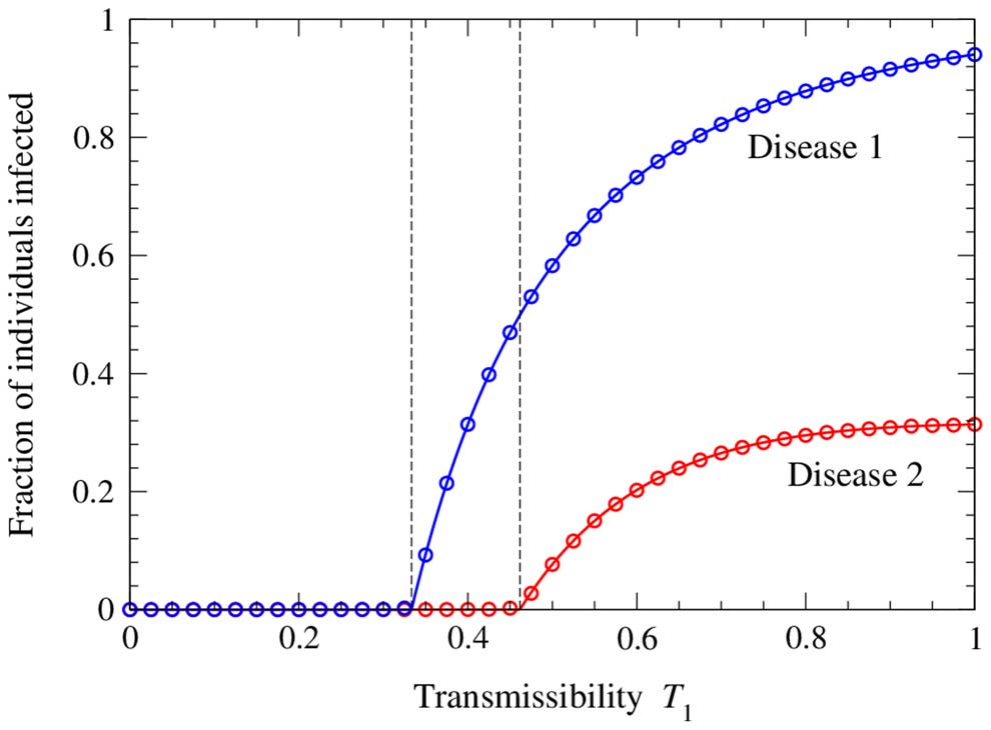
Number of individuals infected with the two diseases on a network with a Poisson degree distribution. The network has mean degree 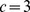 and the transmissibility of the second disease is fixed at 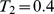, while the transmissibility 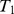 of the first disease is varied. The solid curves show the analytical solutions, Eqs. (7) and (17), while the points show the results of numerical simulations of the model. Each point is an average of simulations on 100 networks of a million nodes each. Error bars are smaller than the points in all cases. The two vertical dashed lines indicate the positions of the epidemic thresholds for the two diseases, from Eqs. (27) and (43).

The epidemic threshold for disease 1 in this case is
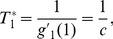
(40)a well known result for a single disease on a Poisson random graph. The epidemic threshold for the second disease is given by Eq. (37). Noting that 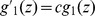, the values of 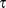 and 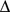 are



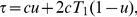
(41)

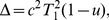
(42)which gives



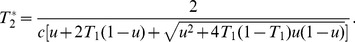
(43)
Equations (19), (25), and (39) cannot be solved exactly, but one can solve them by numerical iteration. We choose suitable starting values for 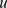, 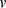 and 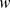 (we find 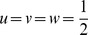 to work well) and iterate the equations to convergence. Figure 2 shows the resulting solutions for the sizes 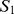 and 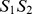 of the two disease outbreaks, as a function of 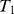 for a network with average degree 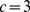 and a fixed value of 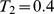. When 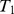 is small we are below the epidemic threshold 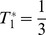 for the first disease, marked by the first vertical line in the figure, and hence neither disease spreads. Above this point the first disease starts to spread but does not, at least at first, infect enough individuals to allow the spread of the second disease. The system goes through a another transition, marked by the second vertical line in the figure, when the size of the first outbreak becomes large enough to support an outbreak of the second. This occurs at the value of 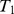 for which Eq. (43) equals 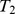.

Thus, in this scenario, it would be possible to eradicate the second disease by either one of two methods: one could take the traditional approach of reducing its transmissibility 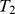 below the threshold value 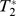, or, alternatively, one could reduce the transmissibility of disease 1 until sufficiently few individuals are infected to allow the spread of disease 2.

Also shown in the figure are numerical results from simulations of the model on computer generated networks with the same Poisson degree distribution. As we can see, agreement between the analytic solution and the numerical results is excellent.

**Figure 3:**
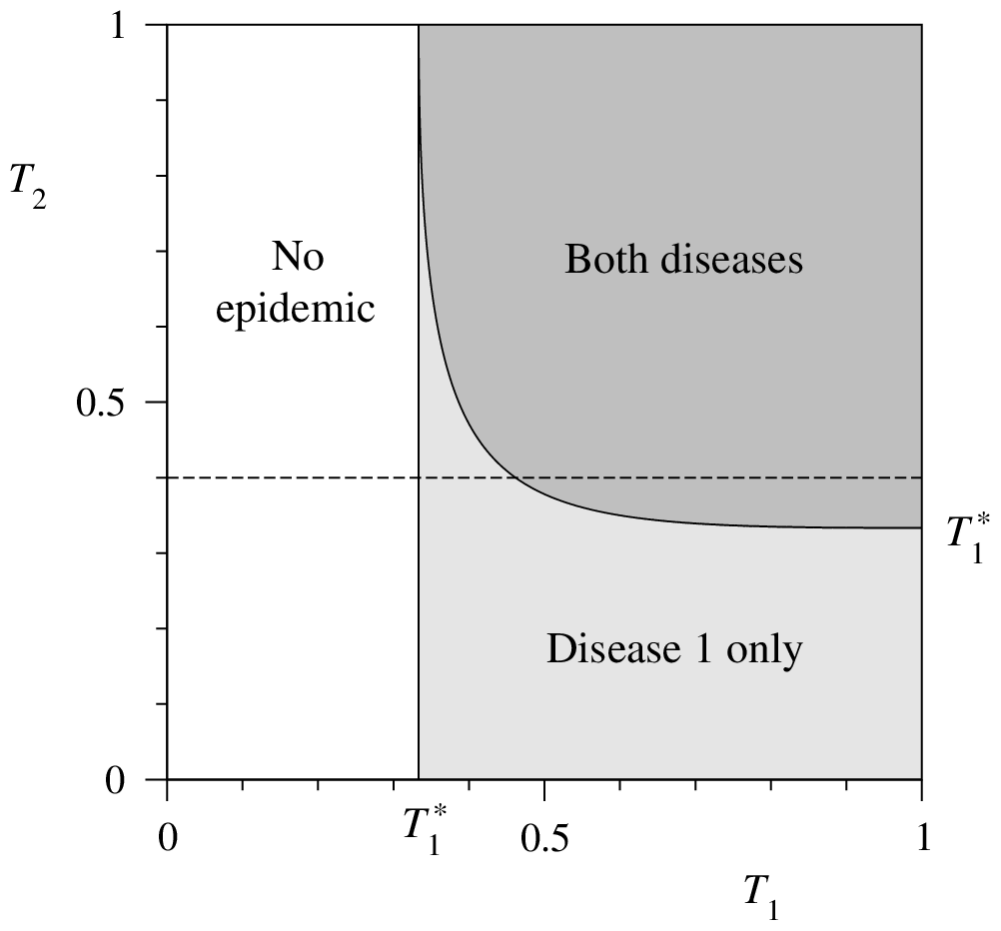
Phase diagram of the model for a network with a Poisson degree distribution with mean degree 3. The horizontal dashed line represents the parameter values used for Fig. 2.

Using the values of 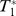 and 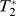 from Eqs. (40) and (43) we can also plot a phase diagram for the model, as in Fig. 3, showing the regions in the 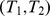 parameter space in which neither, one, or both of the diseases spread. The horizontal dashed line in the figure represents the parameter values used in Fig. 2.

As another example, consider a network with a power-law degree distribution. As pointed out by Pastor-Satorras and Vespignani [12], the epidemic threshold for a single disease on such a network falls at 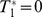 provided the exponent of the power law is less than 3. This means that the disease always produces an epidemic outbreak, no matter how low its transmissibility. From the results above we can show that the same will be true for both diseases in our two-disease coinfection system. The first disease behaves exactly as would a single disease spreading on its own, and hence previous results such as those of Ref. [12] apply and 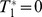. Alternatively, one can evaluate the generating function 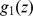 and show that 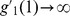 in a power-law network and hence, by Eq. (27), we have 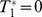. Given that 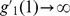, however, we also see that 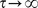 and 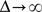 in Eqs. (35) and (36), and hence that 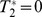 in Eq. (37). In other words, the second disease will also always spread, no matter how low the transmissibility of either the first or second diseases. In this case the second disease cannot be eradicated by lowering either of the transmission probabilities.

The intuitive explanation of this result is that the subnetwork over which the second disease spreads, which consists of those individuals infected with the first disease, also has a power-law tail to its degree distribution; the probability of infection with disease 1 increases with node degree and tends to one in the limit of large degree, so that the degree distribution of infected nodes is the same as that of the network as a whole in the large-degree limit. And it is only the power-law tail that is needed to drive the epidemic threshold to zero–it is not required that the distribution follow a pure power law over its entire domain.

Even though both diseases may spread, however, it is not necessarily the case that many individuals are infected. Indeed the number of individuals infected with disease 1 will necessarily go to zero asymptotically as 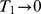, and hence so also will the number infected with disease 2 (which can never exceed the number infected with disease 1).

### Conclusions

In this paper we have studied a simple model of coinfection with two diseases that spread over the same network of contacts. In this model one disease can spread freely through the population, limited only by its probability of transmission, but the second disease can infect only those infected with the first. The result is a system displaying two distinct epidemic thresholds, one occurring when the transmission probability of the first disease reaches a high enough value to support an epidemic outbreak, and the second occurring when the first disease infects a large enough fraction of the population to allow spread of the second disease. Thus, while the first disease can (on a given network) be controlled only by reducing its probability of transmission, the second can be controlled either by reducing transmission or by reducing the number of individuals infected with the first disease.

We have given an analytic solution for the size of both outbreaks and the position of both thresholds on networks generated using the configuration model, for any choice of the degree distribution. The solution is exact in the limit of large network size and shows good agreement with numerical simulations for large but finite networks. We have discussed two specific examples, of a network with a Poisson degree distribution and a network with a power-law degree distribution. In the former case we find a distinct epidemic threshold for the second disease that depends on the transmission probability for the first disease in such a way that the second disease can be controlled or eradicated by reducing either its probability of transition or that of the first disease. In the power-law case, by contrast, we find that the epidemic threshold for both diseases falls at transmission probability zero, so that both will always spread, no matter how low the transmission probabilities are.

A number of questions are unanswered by our analysis. In particular, we have not addressed any dynamical features of the epidemic process, such as the time-scales or rate of growth of the epidemics. And we have considered only the case where the two diseases spread at well separated times. If they were to spread at the same time, it is possible one might see an additional dynamical transition of the kind seen, for example, in [9].

Furthermore our model covers only the case in which infection with the first disease is a necessary condition for infection with the second, and not the more general case where the first disease enhances transmission of the second but is not an absolute requirement. These issues, however, we leave for future work.
